# Konjunkturpolitik — post COVID-19

**DOI:** 10.1007/s10273-020-2688-1

**Published:** 2020-07-18

**Authors:** Überblick

**Keywords:** E32, E62, H12

## Abstract

Die Corona-Krise hat die deutsche Wirtschaft in die tiefste Rezession seit dem Zweiten Weltkrieg gestürzt. Aufgrund der weltweiten Verbreitung sind Lieferketten gestört und der internationale Handel erheblich beeinträchtigt, was die exportabhängige deutsche Wirtschaft besonders stark trifft. Das von der Bundesregierung aufgelegte Programm zur Stützung der Wirtschaft wird von den Autoren in vielen Punkten kritisiert. Einige bewerten vor allem die temporäre Senkung der Umsatzsteuer als eine Maßnahme nach dem Gießkannenprinzip mit geringem Konjunkturimpuls. Sie sehen vor allem die Unternehmen als Leidtragende und Impulsgeber für eine Rückkehr zu einem Wachstum auf Vorkrisen-Niveau. Sie schlagen eine Verbesserung des steuerlichen Umgangs mit Verlusten und eine Senkung der Ertragsteuern vor. Andere legen den Schwerpunkt auf öffentliche Investitionen als Wachstumsmotor und fordern Zukunftsinvestitionen für eine innovative, digitale und klimaneutrale Wirtschaft.

## Abstract (en)

The coronavirus crisis has plunged the German economy into the deepest recession since the Second World War. Due to its global spread, supply chains have been disrupted and international trade has been severely impaired, which has hit the export-dependent German economy particularly hard. The economic stimulus package of the German government is criticized by the authors in many points. Some assess above all the temporary reduction of the value added tax as too general to provide an economic stimulus. They claim that companies suffer most from the pandemic and are the main driving force of potential growth. They propose an improvement in the tax treatment of losses and a reduction in income taxes. Others focus on public investment as a driver of growth and call for future investments for an innovative, digital and climate-neutral economy.

